# Association between blood *Loa loa* microfilarial density and proteinuria levels in a rural area of the Republic of Congo (the MorLo project): a population-based cross-sectional study

**DOI:** 10.1016/S2666-5247(23)00142-8

**Published:** 2023-09

**Authors:** Jérémy T Campillo, Marlhand C Hemilembolo, Sébastien D S Pion, Elodie Lebredonchel, Valentin Dupasquier, Charlotte Boullé, Ludovic G Rancé, Michel Boussinesq, François Missamou, Cédric B Chesnais

**Affiliations:** a TransVIHMI, Université de Montpellier, INSERM Unité 1175, Institut de Recherche pour le Développement, Montpellier, France; b Programme National de Lutte contre l'Onchocercose, Direction de l'Épidémiologie et de la Lutte contre la Maladie, Ministère de la Santé et de la Population, Brazzaville, Republic of the Congo; c Département de Biochimie, Hôpitaux Universitaires Paris Nord Val de Seine, Assistance Publique des Hôpitaux de Paris, Paris, France; d Department of Cardiology, Montpellier University Hospital, Montpellier, France; e Department of Infectious and Tropical Diseases, Montpellier University Hospital, Montpellier, France; f Department of Anesthesiology and Critical Care Medicine, Montpellier University Hospital, Montpellier, France

## Abstract

**Background:**

Case reports have hypothesised that proteinuria, sometimes with glomerulopathy or nephrotic syndromes, might be associated with loiasis. To our knowledge, no study has been done to assess this association. We aimed to investigate the association between *Loa loa* microfilariae burden and proteinuria.

**Methods:**

We did a cross-sectional study between May 16, 2022, and June 11, 2022, to assess the relationship between *Loa loa* microfilaraemia densities and proteinuria in a rural area of the Republic of Congo. We included all consenting adults living in the target area at study commencement who had *L loa* microfilarial densities greater than 500 microfilariae per mL during previous screening for a clinical trial in 2019. This study is part of the MorLo project, and used the project's study population of individuals aged 18 years or older who were living near Sibiti. For each microfilaraemic individual, two individuals without *L loa* microfilarial densities matched on age, sex, and place of residence were included. The association between proteinuria (assessed by dipstick) and *L loa* microfilarial densities, age, and sex was assessed using an unconstrained ordinal regression model since the parallel-lines assumption was violated for microfilarial densities.

**Findings:**

991 participants were included, of whom 342 (35%) were *L loa* microfilaraemic. The prevalence of microfilaraemia was 38% (122 of 325) among individuals with trace proteinuria (<300 mg/24 h), 51% (45 of 89) among individuals with light proteinuria (300 mg to 1 g/24 h), and 71% (15 of 21) among individuals with high proteinuria (>1 g/24 h). Individuals with high proteinuria had significantly higher *L loa* microfilarial densities (p<0·0001): mean microfilariae per mL were 1595 (SD 4960) among individuals with no proteinuria, 2691 (7982) for those with trace proteinuria, 3833 (9878) for those with light proteinuria, and 13 541 (20 118) for those with high proteinuria. Individuals with 5000–14 999 microfilariae per mL and individuals with 15 000 microfilariae per mL or greater were, respectively, 5·39 and 20·49 times more likely to have a high proteinuria than individuals with no microfilaraemia.

**Interpretation:**

The risk of proteinuria increases with *L loa* microfilaraemia. Further studies are needed to identify renal disorders (eg, tubulopathies, glomerulopathies, or nephrotic syndromes) responsible for loiasis-related proteinuria.

**Funding:**

European Research Council, MorLo project.

**Translation:**

For the French translation of the abstract see Supplementary Materials section.

## Introduction

Loiasis is a parasitic disease caused by *Loa loa*, a filarial nematode transmitted from human to human by tabanids (*Chrysops* spp). *L loa* is transmitted only in Africa, from southeast Benin to South Sudan and from Chad to Angola. Demographic data suggest that, in total, tens of millions of people are exposed to the parasite and about 15 million are actually infected.^1^

Female worms produce millions of larvae (ie, microfilariae), which circulate in the blood. Individuals with *L loa* can have very high microfilarial densities, sometimes exceeding 100 000 microfilariae per mL of blood. Loiasis is a chronic disease because adult worms can live up to 20 years and the microfilarial densities remain very stable over time if the individual is not treated with diethylcarbamazine or ivermectin.^2^, ^3^, ^4^

Loiasis constitutes an impediment to programmes aimed at eliminating lymphatic filariasis and onchocerciasis, which are based on mass distribution of ivermectin, a drug that can induce a potentially fatal encephalopathy in individuals with a very high *L loa* microfilarial density.^5^

Loiasis is known to cause somewhat benign manifestations, such as pruritus, the subconjunctival migration of the adult worms (ie, eye worm), and transient episodes of angioedema (ie, Calabar swellings). In addition, loiasis can induce complications affecting different organs such as the heart, CNS, spleen, and kidneys.^6^ A retrospective population-based cohort study in Cameroon showed that people with high *L loa* microfilarial densities had a significantly reduced life expectancy and that the risk of premature death might be proportional to an individual's microfilarial densities.^7^

**Research in context**

**Evidence before this study**

Loiasis, a filarial infection caused by *Loa loa* and transmitted by tabanids (*Chrysops* spp) is exclusively endemic in Africa. We searched PubMed for articles published in English, French, Spanish, or Italian from database inception until March 31, 2022. We used the terms “loiasis”, “filariasis”, “*Loa loa*”, “proteinuria”, “haematuria”, “renal”, “kidneys”, “nephrotic”, and “glomerulopathy”. Using the same terms, we also retrieved relevant articles by searching Google Scholar, theses, and national and international reports. Only 15 case reports of renal disorders in individuals infected with *L loa* microfilaraemia were identified, none of which objectively demonstrated the involvement of loiasis in these disorders.

**Added value of this study**

To our knowledge, this is the first study to investigate the association between loaisis and renal disorders. This population-based cross-sectional study, done in the Lékoumou District of the Republic of the Congo, included 991 individuals, matched on sex, age, microfilaraemia status, and village of residence. This study showed that people with microfilaraemia are more likely to have significantly increased proteinuria than individuals with no microfilaraemia, with a strong positive correlation between microfilarial densities and the severity of proteinuria. In addition, we showed that about a third of the proteinuria were due to *L loa* microfilaraemia.

**Implications of all the available evidence**

Our study supports the hypothesis that *L loa* microfilaraemia can lead to proteinuria, suggesting substantial glomerular and interstitial damage. The high burden of proteinuria we observed in our study could represent the reality across the forested area of central Africa. Further studies are needed to elucidate the pathophysiological mechanisms of the parasite on the kidneys. Our study should motivate further clinical investigations of renal function in loaisis-infected individuals such as renal ultrasounds and urinalysis. The use of a prospective cohort to provide temporal evidence for a causal relationship, while adjusting for known factors of renal disorders, could infer an association between loaisis and renal disorders.

Impairment of renal function has been documented in 15 case reports of loiasis but, to our knowledge, no study has been done to assess the possible complications associated with loiasis. The described renal dysfunction include haematuria, proteinuria (or albuminuria), glomerulopathies, and nephrotic syndromes (appendix 2 p 1). Moreover, some studies report that remission of renal disorder was reached after the administration of antifilarial treatment (ie, diethylcarbamazine or ivermectin). Other studies have reported a transient increase in proteinuria and haematuria just after the administration of antifilarial treatment, suggesting that the renal manifestations might be immunologically induced by the lysis of microfilariae and their elimination through the kidneys (appendix 2 p 1).

Proteinuria has also been reported in individuals infected with other filarial species such as *Wuchereria bancrofti, Brugia malayi*, and *Brugia timori* (causing lymphatic filariasis)^8^, ^9^, ^10^ and, to a lesser extent, *Onchocerca volvulus* (onchocerciasis).^11^, ^12^

We report the results of the first cross-sectional study investigating the relationship between proteinuria and *L loa* microfilarial densities.

## Methods

### Study design

In this cross-sectional study, we assessed the relationship between *L loa* microfilarial densities and proteinuria in a rural area of the Republic of Congo. This study is part of the Morbidity due to Loiasis (MorLo) project, which is an international collaborative study designed to assess the prevalence and incidence of *Loa loa*-related organ-specific complications in rural sub-Saharan Africa. We therefore used the MorLo study population of individuals living in 21 villages located near Sibiti, the capital town of the Lékoumou division of the Republic of Congo between May 16, 2022, and June 11, 2022.

This study was approved by the Ethics Committee of the Congolese Foundation for Medical Research (036/CIE/FCRM/2022) and by the Congolese national administrative authorities (376/MSP/CAB/UCPP-21).

All participants provided written informed consent.

### Participants

Participants were recruited from a 2019 clinical trial^13^ that examined for *L loa* microfilaraemia during screening. The inclusion criterion was living in the target area at study commencement. Participants were also aged 18 years or older.

The individuals included will be followed up for 3 years with the instruction to report any health event. Health assessments will also be done annually. This region is endemic for loiasis, not for schistosomiasis, and deworming campaigns are regularly carried out on children to control soil-transmitted helminthiases. Individuals with more than 500 *L loa* microfilariae per mL of blood in 2019 were matched on sex and age (within 5 years), with two individuals living in the same village identified as amicrofilaraemic in 2019.

### Procedures

All participating adults were taken to the filariasis care centre at Sibiti District Hospital (Sibiti, Republic of Congo) where all examinations were done. Participants might have had symptomatic treatments for their loaisis-related health problems (eg, antihistaminics, painkillers, and anti-inflammatories) or non-loasis disorders (eg, antacids and antispasmodics). Sex at birth data were collected by the interviewer when participants were included.

50 μL of blood was collected by fingerprick between 1000 h and 1600 h pm from each participant to prepare a thick blood smear. All Giemsa-stained thick blood smears were examined under a microscope by two experienced technicians to count the *L loa* microfilariae. The arithmetic means of the microfilarial densities measured at the two readings were used for the analyses and the results were expressed in microfilariae per mL.

Following instruction by a nurse, each participant provided one morning midstream urine specimen. A dipstick (Reactif urine analysis strips, Nal von Minden, Moers) urinalysis was immediately done to detect proteinuria. All dipsticks were read by a single physician who did a second test to confirm the result if the first dipstick result was positive. Dipstick results are expressed as follows: negative, traces (corresponding to <300 mg protein per 24 h), positive level 1 (300 mg to 1 g protein per 24 h), positive level 2 (1–3 g protein per 24 h), or positive level 3 (>3 g protein per 24 h). The physician in charge of reading the urine strips did not know the microfilaraemic status of each individual. Despite schistosomiasis not being endemic in the area, any individual with haematuria underwent microscopic examination of urine for *Schistosoma* spp eggs and serological testing (Schistosoma ICT IgG-IgM. LD BioDiagnostic, Lyon). Soil-transmitted helminth infections were also detected by microscopic examination of stool specimens on a proportion (all who volunteered) of the study population. Two thick smears were prepared according to the Kato-Katz method for each stool sample.

### Outcomes

The primary outcome was to evaluate the association between *L loa* microfilarial densities and level of proteinuria (negative, traces, and positivity levels 1–3), evaluated by dipstick. The secondary outcome was to assess the relationship between *L loa* microfilarial densities and level of proteinuria, adjusted for sex and age.

### Statistical analysis

Variables of interest were sex, age, *L loa* microfilaraemia status (ie, absent or present), *L loa* microfilarial densities (four categories: 0 microfilariae per mL, 1–4999 microfilariae per mL, 5000–14 999 microfilariae per mL and ≥15 000 microfilariae per mL), and level of proteinuria (negative, trace, lightly positive [ie, 1+], or highly positive [ie, >1+]). To improve the quality of our statistical models, *L loa* microfilarial densities were analysed based on the density categories and not with the IQRs for convergence issues. All analyses were done on a complete dataset, with no missing values observed for any of the variables included in the study.

Characteristics (ie, age, sex, and *L loa* microfilarial densities) of participants were described and compared by level of proteinuria. χ^2^ test (with Yates correction if one of the expected counts was lower than 5) was done for categorical variables and Kruskal-Wallis rank test was done for continuous variables. Ordinal regression models were used to assess the relationship between proteinuria and *L loa* microfilarial densities. The coefficient for *L loa* microfilarial densities that describes the relationship between no proteinuria versus all higher proteinuria categories was not the same as the coefficient that describes the relationship between trace proteinuria and all higher proteinuria categories, meaning that the *L Loa* microfilarial densities categorical variable was violated for the parallel-lines assumption (or proportional odds assumption) necessary for classic ordinal models. Due to this violation, a partial proportional odds model was fitted with a constrained coefficient for age and sex and an unconstrained coefficient for *L loa* microfilarial densities.^14^ Therefore, the model compares and contrasts the category no proteinuria with the three other levels of proteinuria; the combined categories of no proteinuria and trace proteinuria with light proteinuria and high proteinuria; and the combined no proteinuria, trace proteinuria, and light proteinuria with high proteinuria. Therefore, positive coefficients indicate that higher *L loa* microfilarial densities increase the likelihood that individuals will be in a higher category of proteinuria than the reference, whereas negative coefficients indicate that higher *L loa* microfilarial densities are associated with a higher likelihood of proteinuria in the reference or a lower category. From this partial proportional odds model, we estimated the probabilities of different levels of proteinuria for each *L loa* microfilarial density category.

To quantify the effect of *L loa* microfilaraemia on proteinuria, the population attributable fraction (ie, the proportion of the proteinuria burden that could be eliminated if *L loa* microfilariae was eliminated) was calculated from a saturated model including age, sex, and *L loa* status (as a binary variable).^15^

Finally, using *L loa* microfilarial densities as an indicator of proteinuria, we estimated the optimal cutoff value to explain the shift from one level of proteinuria to another. The Youden method was used with bootstrapping (500 repetitions).^16^ The optimal cutoff values were identified using sensitivity, specificity, and the area under the receiver operating characteristics (ROC) curve.

All statistical analyses were done using Stata 15.

### Role of the funding source

The funders of the study had no role in study design, data collection, data analysis, data interpretation, or writing of the report.

## Results

The final study population included 990 participants aged 18–88 years. Among the 990 participants (619 men and 371 women), one participant decided to leave the study (figure). A total of 342 (35%) individuals were *L loa* microfilaraemic. 325 (33%) individuals had trace proteinuria, 89 (9%) had light proteinuria, and 21 (2%) had high proteinuria (table 1). Sex was not associated with level of proteinuria (p=0·18). As age increased, people were more likely to have higher levels of proteinuria (p<0·0001). Individuals who were microfilaremic had proteinuria more frequently than individuals who were amicrofilaraemic (p<0·0001). Mean and median *L loa* microfilarial densities significantly increased with the level of proteinuria (p<0·0001) and individuals with more than 5000 microfilariae per mL were more likely to have high proteinuria (p<0·0001). 83 individuals (8%) had indicative haematuria (1+ blood in urine or greater). 31 (28%) of the 110 individuals with at least light proteinuria also had indicative haematuria in the study. No schistosomiasis was found in the individuals with haematuria. No association between haematuria and loiasis was found (appendix 2 p 3). 58 (6%) of all 991 individuals had stool examinations for soil-transmitted helminths infections. Of them, no individuals had hookworm infections, 36 (62%) had *Ascaris* spp infections, and 39 (67%) had *Trichuris* spp infections (appendix 2 p 3).

**Figure:**
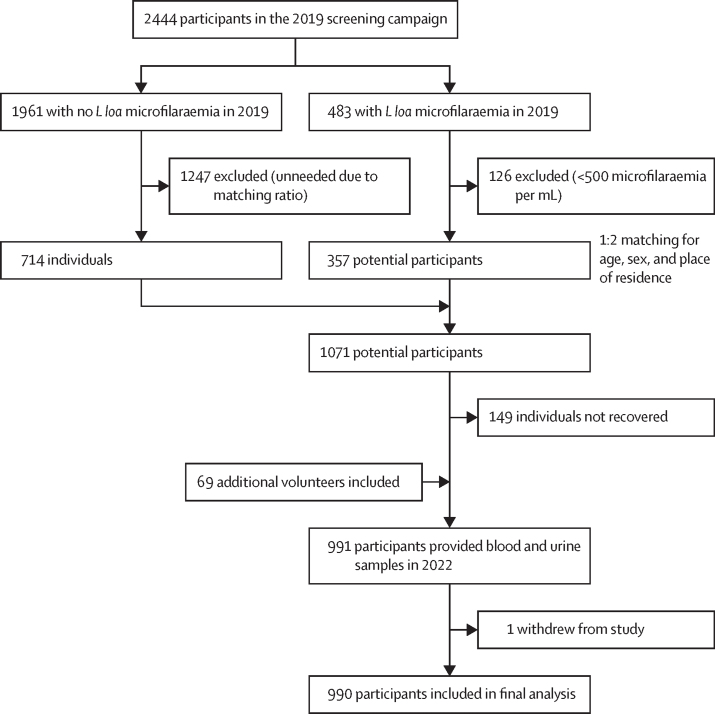
Trial profile

**Table 1:** Distribution of the main variables of interest according to proteinuria status

		**Total (n=990)**	**Level of proteinuria**				p value*
			Negative (n=555)	Traces (n=325)	Lightly positive (n=89)	Highly positive (n=21)	
Sex		..	..	..	..	..	0·18
	Male	619 (63%)	333 (60%)	218 (67%)	54 (61%)	15 (71%)	..
	Female	371 (38%)	222 (40%)	107 (33%)	35 (39%)	6 (29%)	..
Age (years)		50·8 (14·8)	48·9 (14·6)	52·0 (14·6)	57·2 (14·3)	54·7 (17·0)	<0·0001
Positive *Loa loa* microfilarial density status		342 (35%)	160 (29%)	122 (38%)	45 (51%)	15 (71%)	<0·0001
*Loa loa* microfilarial density (microfilariae per mL)		..	..	..	..	..	<0·0001
	Arithmetic mean (SD)	2409·4 (7398·5)	1594·6 (4960·9)	2691·4 (7982·4)	3833·9 (9878·3)	13 540·9 (20 117·8)	..
	Median (IQR)	0 (0–540)	0 (0–140)	0 (0–730)	20 (0–3130)	8760 (0–19710)	..
	Geometric mean (95% CI)	1789 (1443–2218)	1581 (1164–2149)	1761 (1221–2539)	1832 (980–3425)	7063 (2445–20 401)	..
*Loa loa* microfilarial density categories		..	..	..	..	..	<0·0001
	0 microfilariae per mL	648 (66%)	395 (71%)	203 (63%)	44 (49%)	6 (29%)	..
	1–4999 microfilariae per mL	217 (22%)	105 (19%)	80 (25%)	28 (32%)	4 (19%)	..
	5000–14 999 microfilariae per mL	82 (8%)	41 (7%)	26 (8%)	11 (12%)	4 (19%)	..
	≥15 000 microfilariae per mL	43 (4%)	14 (3%)	16 (5%)	6 (7%)	7 (33%)	..
IQR of *Loa loa* microfilarial density		..	..	..	..	..	<0·0001
	0 microfilariae per mL	648 (66%)	395 (71%)	203 (63%)	44 (49%)	6 (29%)	..
	1–499 microfilariae per mL	91 (9%)	43 (9%)	33 (10%)	12 (14%)	3 (14%)	..
	500–2499 microfilariae per mL	81 (8%)	39 (7%)	32 (10%)	9 (10%)	1 (5%)	..
	2500–9999 microfilariae per mL	94 (10%)	48 (9%)	29 (9%)	14 (16%)	3 (14%)	..
	≥10 000 microfilariae per mL	76 (8%)	30 (5%)	28 (9%)	10 (11%)	8 (38%)	..

Data are n (%) or mean (SD) unless otherwise specified. Percentages might not sum to 100 because of rounding. Lightly positive is 1+ proteinuria. Highly positive is >1+ proteinuria.

* χ^2^ for categorical variables (with Yates correction if 1n_ij_ <5) and Kruskal-Wallis rank test for continuous variables.

Parallel-lines assumption was not violated for age (p=0·15) and sex (p=0·34) but was violated for *L loa* microfilaraemia (p=0·044). Therefore, for the ordered model, age and sex coefficients were constrained to have the same value across equations whereas *L loa* microfilarial density was unconstrained across equations (table 2). The odds ratio for age was 1·02 (95% CI 1·01–1·03, p<0·0001) and for sex was 1·34 (1·03–1·74, p=0·03), with female as the reference value; indicating that older age and being male were significantly associated with higher levels of proteinuria. Individuals who were microfilaraemic were more likely to have a high level of proteinuria than individuals who were amicrofilaraemic. A positive association was identified: individuals with the highest *L loa* microfilarial densities (ie, 5000–14 999 microfilariae per mL and ≥15 000 microfilariae per mL) are more likely to have a high proteinuria than those with low microfilarial densities (1–4999 microfilariae per mL) who are more likely to have light proteinuria.

**Table 2:** Odds ratios from unconstrained partial ordered logit models

	**Negative *vs* traces, lightly positive, and highly positive**	**Negative and traces *vs* lightly positive and highly positive**	**Negative, traces, and lightly positive *vs* highly positive**
0 microfilariae per mL	Ref (1)	Ref (1)	Ref (1)
1–4999 microfilariae per mL	1·61 (1·17–2·03), p=0·0030	1·97 (1·22–3·18), p=0·0051	1·90 (0·52–6·79), p=0·33
5000–14 999 microfilariae per mL	1·54 (0·97–2·45), p=0·07	2·65 (1·41–4·99), p=0·0025	5·39 (1·48–19·5), p=0·010
≥15 000 microfilariae per mL	3·13 (1·61–6·10), p=0·0008	5·10 (2·48–10·43), p<0·0001	20·49 (6·52–64·37), p<0·0001

Data are odds ratio (95% CI), p value.

For the saturated unconstrained partial ordered model, the same positive correlation is observable: individuals with no microfilariae have a 0·9% risk of having high proteinuria, while the risk is 1·8% for those with 1–4999 microfilariae per mL, 4·9% for those with 5000–14 999 microfilariae per mL, and 16·4% for those with 15 000 or greater microfilariae per mL (table 3).

**Table 3:** Probability of having proteinuria according to microfilarial density

	**Negative**	**Trace**	**Positive level 1**	**Positive levels 2 and 3**
0 microfilariae per mL	60·8% (8·4)	31·4% (5·9)	6·8% (2·3)	0·9% (0·3)
1–4999 microfilariae per mL	48·2% (8·7)	37·0% (4·5)	12·9% (3·7)	1·8% (0·6)
5000–14 999 microfilariae per mL	49·9% (8·2)	31·8% (3·2)	13·5% (3·4)	4·9% (1·6)
≥15 000 microfilariae per mL	32·7% (7·0)	36·9% (1·0)	13·9% (2·4)	16·4% (4·5)

Data are probability (SE). Trace proteinuria refers to <300 mg per 24 h. Positive level 1 refers to proteinuria of >300 mg protein per 24 h. Positive level 2 refers to >1 g protein per 24 h. Positive level 3 refers to >3 g protein per 24 h.

The population attributable fractions of proteinuria associated with *L loa* microfilaraemia was 10·2% (95% CI 4·9–15·3) when proteinuria was defined as trace or greater and 29·9% (15·0–42·2) when proteinuria was defined as light or greater.

The optimal *L loa* microfilarial density cutoff was identified as 8670 microfilariae per mL. Discrimination of no proteinuria from trace proteinuria or greater had a sensitivity of 0·29, a specificity of 0·80, and an area under the ROC curve of 0·54. Discrimination of light proteinuria and greater from no or trace proteinuria had a sensitivity of 0·37, a specificity of 0·78, and an area under ROC curve of 0·57. Discrimination of high proteinuria from lower levels had a sensitivity of 0·73, a specificity of 0·78, and an area under ROC curve of 0·76.

## Discussion

Using a cross-sectional design, we describe, for the first time, an association between the presence of *L loa* microfilaraemia and proteinuria with a positive correlation between microfilarial densities and the severity of proteinuria. These findings are consistent with multiple case reports of renal manifestations in individuals with loiasis (appendix 2 p 1). Increased proteinuria was also reported for individuals infected with *Onchocerca volvulus*^11^, ^12^ or with lymphatic filariasis,^8^, ^9^, ^10^ and for animals infected with *Dirofilaria immitis.*^17^, ^18^ The only epidemiological study that assessed the relationship between filariasis and proteinuria at a community level, focused on onchocerciasis, did not find a statistically significant relationship between *O volvulus* microfilaraemia and proteinuria.^19^

Among individuals with renal disorders for whom involvement of *L loa* was suspected, only 11 had a renal biopsy. These biopsies revealed various types of lesions: membranoproliferative glomerulonephritis, focal segmental glomerulosclerosis, and focal interstitial inflammation (appendix 2 p 1). In all these cases, proteinuria was reported. If we consider more than 300 mg protein per 24 h to be pathological proteinuria (light proteinuria or higher), individuals with 5000–14 999 microfilariae per mL and with more than 15 000 microfilariae per mL were, respectively, 5·39 and 20·49 times more likely to have pathological proteinuria than individuals without microfilariae. Moreover, the calculated population attributable fractions were high, especially for pathological proteinuria with a population attributable fraction of 29·9%, meaning that, in our study population, 29·9% of the burden of proteinuria might be eliminated by eliminating *L loa* microfilariae, all else being equal. Since population attributable fractions mainly depend on the prevalence of exposure, the population attributable fractions must be interpreted in a given population and with caution. However, the *L loa* microfilariae prevalence in this population is representative of what is observed across regions of central Africa where loiasis is endemic, meaning that the high burden of proteinuria possibly related to loiasis we observed in our population might represent the reality across forested areas of central Africa.

The pathophysiological mechanisms through which loiasis can induce renal manifestations are unclear but might involve multiple mechanisms: a direct effect, depending on the microfilarial loads, and an indirect, possibly immuno-mediated effect. Multiple case reports noted the presence of *L loa* microfilariae in urine and a transient worsening of renal symptoms following antifilarial therapy, followed by improvement or even complete recovery (appendix 2 p 1). These observations support the hypothesis that the physical presence of *L loa* microfilariae (diameter approximately 7 μm) in the glomeruli and tubules (diameter approximately 30 μm) leads to the establishment of renal manifestations such as proteinuria.^20^ The observed dose effect between *Loa loa* microfilarial densities and proteinuria levels might illustrate this mechanism. The indirect mechanism might be mediated by the liberation of filarial antigens^21^ and the deposits of immune complexes,^22^, ^23^, ^24^ possibly associated with a chronic biological inflammation. Since high proteinuria has been found in individuals with low microfilarial densities (<5000 microfilariae per mL) and the optimal cutoff to predict high proteinuria was calculated as 8670 microfilariae per mL, this effect might be not dependent on individual *Loa loa* microfilarial densities.

This study has multiple limitations. We assessed the presence of proteinuria only with dipsticks, which do not allow the detection of proteins with low molecular weight and which can be positive in the case of urinary tract infections. However, at the time proteinuria was assessed, all participants were examined by a physician and no signs of urinary tract infections were observed or reported by the participants. Moreover, in the case of positive proteinuria, a second urine dipstick was systematically performed to confirm the result, and no discrepancy was observed between the two consecutive tests. Nevertheless, proteinuria due to proteins with low molecular weight cannot be excluded, and the underlying mechanism could still be linked to glomerulopathy or to high-grade tubulopathy. This study took a cross-sectional design, which cannot establish causal relationships meaning that the results should be interpreted with caution. To gain a deeper understanding of the factors contributing to significant proteinuria in this population, further research using case-control or cohort studies might be necessary. Nevertheless, given the very high and significant odds ratios found in this study, the association found is highly unlikely to be due to chance or unidentified risk factors.

The results of this study support the existence of *L loa*-induced proteinuria. Further investigations are needed to assess renal function (and classifications of renal failure, if necessary). Adjustments for possible risk factors of proteinuria and renal disorders (eg, diabetes or hypertension) should be included in these next steps, and, ideally, a prospective cohort to provide the temporal criteria for a causal relationship.

Our results should also be compared with estimates of the prevalence of all-cause kidney disease in rural areas of central Africa with and without loiasis endemicity. However, data is very scarce.^25^ In a cross-sectional study done in six regions of the world, the prevalence of chronic kidney disease in the general population was estimated at 14·3% and estimates varied greatly by country and presence of well known risk factors (eg, diabetes, systemic hypertension, and HIV with HIV-associated nethropathy).^26^ In a cross-sectional study done in Cameroon, the authors evaluated the prevalence of renal failure and albuminuria (assessed by dipstick) in rural and urban areas of the Dschang district, where loaisis is endemic. Renal failure prevalence was estimated at 24·1% and proteinuria prevalence was 23·8%. Prevalence of proteinuria at greater than 300 mg protein per 24 h (corresponding to 1+ proteinuria or greater) was 23·8% in rural areas compared with 8·4% in urban areas.^27^ This high prevalence in rural areas is not fully explained by the most common causes or the level of loiasis endemicity (the history of eye worm is estimated at 20–30%).^1^ In our study population, *L loa* microfilaraemia prevalence (34·5%) and proteinuria prevalence (43·9%, with a prevalence of 11·1% for proteinuria >300 mg protein per 24 h) were higher than the study in Cameroon, and further studies should be done to better understand the various causes of proteinuria in loiasis-endemic areas.

Our results provide the rationale for further investigation of the effect of *L loa* infection on renal function to improve understanding of the pathophysiological mechanisms of loiasis in the establishment of renal disorders.

**This online publication has been corrected. The corrected version first appeared at thelancet.com/microbe on July 26, 2023**

## Data sharing

Data cannot be shared directly by the authors because of data protection regulation. Data are accessible to authorised researchers after signed data access agreement. Requests for access should be sent to the corresponding author (JTC).

## Declaration of interests

CB is the recipient of a grant from the Bettencourt-Schueller Foundation (CCU-AH-INSERM-Bettencourt). All other authors declare no competing interests.

## Supplementary Materials

French translation of the abstract
