# Supplementary material for: Association between blood Loa loa microfilarial density and proteinuria levels in a rural area of the Republic of Congo (the MorLo project): a population-based cross-sectional study
Source: Lancet Microbe. 2023 Sep;4(9):e704–10. doi: 10.1016/S2666-5247(23)00142-8 (PMC10469262; doi:10.1016/S2666-5247(23)00142-8)
Supplement: French translation of the abstract [file mmc1.pdf]

THE LANCET
Microbe

Supplementary appendix 1

This translation in French was submitted by the authors and we reproduce it as supplied. It has not been peer reviewed. *The Lancet*'s editorial processes have only been applied to the original in English, which should serve as reference for this manuscript.

Cette traduction en français a été proposée par les auteurs et nous l'avons reproduite telle quelle. Elle n'a pas été examinée par des pairs. Les processus éditoriaux du *Lancet* n'ont été appliqués qu'à l'original en anglais et c'est cette version qui doit servir de référence pour ce manuscrit.

This online publication has been corrected. The corrected version first appeared at thelancet.com/microbe on July 26, 2023.

Supplement to: Campillo JT, Hemilembolo MC, Pion SDS, et al. Association between blood *Loa loa* microfilarial density and proteinuria levels in a rural area of the Republic of Congo (MorLo): a population-based cross-sectional study. *Lancet Microbe* 2023; published online July 19. https://doi.org/10.1016/S2666-5247(23)00142-8.

Résumé (en français)

Contexte. Des rapports de cas ont émis l'hypothèse que la protéinurie, parfois accompagnée de glomérulopathie ou de syndromes néphrotiques, pourrait être associée à la loase. À notre connaissance, aucune étude n'a été réalisée pour évaluer cette association. Nous avons cherché à évaluer l'association entre la charge microfilarienne en *Loa loa* et la protéinurie.

Méthodes. Nous avons réalisé une étude transversale entre le 16 mai et le 11 juin 2022 afin d'évaluer la relation entre la charge microfilarienne en *Loa loa* et la protéinurie en zone rurale de la république du Congo. Nous avons inclus tous les adultes consentants, vivant dans la zone d'étude au départ de la recherche et ayant une charge microfilarienne en *Loa loa* supérieure à 500 microfilaires par millilitre de sang durant un dépistage ayant eu lieu en 2019 dans le cadre d'un essai clinique. Cette étude fait partie du projet MorLo et a utilisé la population du projet, à savoir les personnes âgées de 18 ans ou plus qui vivaient près de Sibiti. Pour chaque individu microfilarémique inclus, deux individus sans microfilarémie ont été appariés sur l'âge, le sexe et le lieu de résidence. L'association entre la protéinurie (évaluée par bandelette réactive urinaire) et les densités microfilariennes en *Loa loa*, l'âge et le sexe a été évaluée à l'aide d'un modèle de régression ordinale non contraint, car l'hypothèse des lignes parallèles n'était pas respectée pour les densités microfilariennes.

Résultats. 991 participants ont été inclus dont 342 (35%) étaient microfilarémiques pour *Loa loa*. La prévalence de la microfilarémie était de 38 % (122 sur 325) chez les personnes présentant une protéinurie à l'état de traces (<300 mg/24 h), de 51 % (45 sur 89) chez les personnes présentant une protéinurie légère (300 mg à 1 g/24 h) et de 71 % (15 sur 21) chez les personnes présentant une protéinurie importante (>1 g/24 h). Les individus présentant une protéinurie importante avaient des densités microfilariennes significativement plus élevées (p<0·0001) : la moyenne des microfilarémies par ml était de 1595 (SD 4960) chez les individus sans protéinurie, de 2691 (7982) chez ceux présentant une protéinurie à l'état de traces, de 3833 (9878) chez ceux présentant une protéinurie légère et de 13 541 (20 118) chez ceux présentant une protéinurie importante. Les individus ayant entre 5000 et 14 999 microfilaires par ml et les individus avec 15 000 microfilaires par ml ou plus étaient, respectivement, 5,39 et 20,49 fois plus susceptibles d'avoir une protéinurie importante que les individus sans microfilarémie.

Interprétation. Le risque de protéinurie augmente avec la microfilarémie *de L loa*. Des études complémentaires sont nécessaires pour identifier les troubles rénaux (tubulopathies, glomérulopathies ou syndromes néphrotiques) responsables de la protéinurie liée à la loase.

Funding European Research Council, MorLo project.
