# Supplementary material for: Association between blood Loa loa microfilarial density and proteinuria levels in a rural area of the Republic of Congo (the MorLo project): a population-based cross-sectional study
Source: Lancet Microbe. 2023 Sep;4(9):e704–10. doi: 10.1016/S2666-5247(23)00142-8 (PMC10469262; doi:10.1016/S2666-5247(23)00142-8)
Supplement: Supplementary appendix 2 [file mmc2.pdf]

THE LANCET
Microbe

Supplementary appendix 2

This appendix formed part of the original submission and has been peer reviewed. We post it as supplied by the authors.

Supplement to: Campillo JT, Hemilembolo MC, Pion SDS, et al. Association between blood *Loa loa* microfilarial density and proteinuria levels in a rural area of the Republic of Congo (MorLo): a population-based cross-sectional study. *Lancet Microbe* 2023; published online July 19. https://doi.org/10.1016/S2666-5247(23)00142-8.

Appendix.

Table 1. References describing renal dysfunction or abnormalities in individuals with loiasis

**Renal dysfunction or abnormalities**	**References**
Hematuria	- Gentilini M, Domart A, Brumpt L, Hazard J, le Quintrec Y. [Filariose à *Loa loa* et protéinurie]. *Bulletin de la Société de pathologie exotique* 1963; **56**: 230–40.<br>- Fritel D, Bariety J, Gentilini M. [Loase et néphropathie. Etude anatomoclinique de trois cas]. *Med Afr Noire* 1970; **1**: 13–5.<br>- Barierty J, Barbier M, Laigre M-C, *et al.* [Protéinurie et loase. Etude histologique, optique et électronique d'un cas]. *Société médicale des hôpitaux de Paris* 1967; **118**: 1015–25.<br>- Ameur A, Haouri M, Touiti D, Beddouch A, Oukheira H. [Hématurie et filariose. Aspects pathogéniques: à propos d'une observation]. *Ann Urol (Paris)* 2000; **34**: 110–1.
Proteinuria (or albuminuria)	- Gentilini M, Domart A, Brumpt L, Hazard J, le Quintrec Y. [Filariose à *Loa loa* et protéinurie]. *Bulletin de la Société de pathologie exotique* 1963; **56**: 230–40.<br>- Fritel D, Bariety J, Gentilini M. [Loase et néphropathie. Etude anatomoclinique de trois cas]. *Med Afr Noire* 1970; **1**: 13–5.<br>- Barierty J, Barbier M, Laigre M-C, *et al.* [Protéinurie et loase. Etude histologique, optique et électronique d'un cas]. *Société médicale des hôpitaux de Paris* 1967; **118**: 1015–25.<br>- Schneider J. [Problèmes diagnostiques et thérapeutiques de médecine tropicale dans la pratique médicale courante en France]. *Bulletin de la société de pathologie exotique* 1964; **57**: 719–65.<br>- Zuidema PJ. Renal changes in loiasis. *Folia Med Neerl* 1971; **14**: 168–72.<br>- Janssens PG, van Bogaert L, Tverdy G, Wanson M. [Réflexions sur le sort des microfilaires de *Loa loa* dans l'organisme humain parasité. Manifestations viscérales provoquées par leur infiltration dans les tissus]. *Bulletin de la Société de pathologie exotique* 1958; **4**: 680–93.<br>- Arborio M, Schill H, Neveux Y, *et al.* [Néphropathie et filariose à *Loa loa* traitée par ivermectine]. Thérapeutiques antiparasitaires : communications. 1995; **55**
Glomerulopathies	- Fritel D, Bariety J, Gentilini M. [Loase et néphropathie. Etude anatomoclinique de trois cas]. *Med Afr Noire* 1970; **1**: 13–5.<br>- Pillay VKG, Kirch E, Kurtzam NA. Glomerulopathy associated with filarial loiasis. *JAMA* 1973; **225**: 179–179.<br>- Pakasa NM, Nseka NM, Nyimi LM. Secondary collapsing glomerulopathy associated with *Loa loa* filariasis. *American Journal of Kidney Diseases* 1997; **30**: 836–9.<br>- Pérez Pérez AJ, Sobrado J, Cigarrán S, Valdés R, González L, Courel M. [Filariasis *loa-loa* como causa de uremia terminal]. *Nefrologia* 1987; **7**: 183–5.<br>- Malik STA, McHugh M, Morley AR, Ngu J, Qureshi M, Wilkinson R. Filariasis (*Loa loa*) associated with membranous glomerulonephritis: demonstration of filarial antigen. *Kidney interactions* 1981; **20**: 157–157.<br>- Lukiana T, Mandina M, Situakibanza NH, *et al.* A possible case of spontaneous *Loa loa* encephalopathy associated with a glomerulopathy. *Filaria J* 2006; **5**.<br>- Katner H, Eugene Beyt B, Krotoski WA. Loiasis and renal failure. *South Med J* 1984; **77**: 907–8.<br>- Adebajol A 0, Akinsola' A, Maizels3 RM, Cawston' TE, Haziernan' BL. Rheumatoid factor and rheumatoid factor isotypes in loiasis with and without accompanying glomerulonephritis. *Transactions of the royal society of tropical medecine and hygiene* 1992; **86**: 667–9.<br>- Abel L, Joly V, Yeni P, Carbon C. Apheresis in the management of loiasis with high microfilariaemia and renal disease. *Br Med J* 1986; **292**: 24–5.
Nephrotic syndromes	- Stephens L, Peat D, Chiodini P. Nephrotic syndrome due to loiasis following a tropical adventure holiday: a case report and review of the literature. *Clin Nephrol* 2001; **56**: 247–50.<br>- Pereda PiA, Marcos GR, Plaza MV, Gonzales JC, Garcia EG. Complete remission of loiasis-associated nephrotic syndrome with collapsing glomerulopathy after diethilcarbamazine treatment. *Enf Emerg* 2010; **12**: 155–8.
Remission of renal disorder after administration of antifilarial treatment	- Pereda PiA, Marcos GR, Plaza MV, Gonzales JC, Garcia EG. Complete remission of loiasis-associated nephrotic syndrome with collapsing glomerulopathy after diethilcarbamazine treatment. *Enf Emerg* 2010; **12**: 155–8.<br>- Hall C, Stephens L, Peat D, Chiodini P. Nephrotic syndrome due to loiasis following a tropical adventure holiday: a case report and review of the literature. *Clin Nephrol* 2001; **56**: 247–50.<br>- Zuidema PJ. Renal changes in loiasis. *Folia Med Neerl* 1971; **14**: 168–72.<br>- Janssens PG, van Bogaert L, Tverdy G, Wanson M. [Réflexions sur le sort des microfilaires de *Loa loa* dans l'organisme humain parasité. Manifestations viscérales provoquées par leur infiltration dans les tissus]. *Bulletin de la Société de pathologie exotique* 1958; **4**: 680–93.<br>- Fritel D, Bariety J, Gentilini M. [Loase et néphropathie. Etude anatomoclinique de trois cas]. *Med Afr Noire* 1970; **1**: 13–5.<br>- Abel L, Joly V, Yeni P, Carbon C. Apheresis in the management of loiasis with high microfilariaemia and renal disease. *Br Med J* 1986; **292**: 24–5.
Transient increase in renal disorder after	- Fritel D, Bariety J, Gentilini M. [Loase et néphropathie. Etude anatomoclinique de trois cas]. *Med Afr Noire* 1970; **1**: 13–5.<br>- Cruel T, Arborio M, Schill H, *et al.* [Néphropathie et filariose à *Loa loa*. A propos d'un cas de réaction adverse à la prise d'ivermectine]. *Bulletin de la Société de pathologie exotique* 1997; **90**: 179–81.

administration of antifilarial treatment	- Katner H, Eugene Beyt B, Krotoski WA. Loiasis and renal failure. *South Med J* 1984; **77**: 907–8.<br>- Gentilini M, Domart A, Brumpt L, Hazard J, le Quintrec Y. [Filariose à *Loa loa* et protéinurie]. *Bulletin de la Société de pathologie exotique* 1963; **56**: 230–40.<br>- Barierty J, Barbier M, Laigre M-C, *et al.* [Protéinurie et loase. Etude histologique, optique et électronique d’un cas]. *Société médicale des hôpitaux de Paris* 1967; **118**: 1015–25.<br>- Arborio M, Schill H, Neveux Y, *et al.* [Néphropathie et filariose à *Loa loa* traitée par ivermectine]. Thérapeutiques antiparasitaires : communications. 1995; **55**.
Abnormal renal biopsy	- Pillay VKG, Kirch E, Kurtzam NA. Glomerulopathy associated with filarial loiasis. *JAMA* 1973; **225**: 179–179.<br>- Pakasa NM, Nseka NM, Nyimi LM. Secondary collapsing glomerulopathy associated with *Loa loa* filariasis. *American Journal of Kidney Diseases* 1997; **30**: 836–9.<br>- Hall C, Stephens L, Peat D, Chiodini P. Nephrotic syndrome due to loiasis following a tropical adventure holiday: a case report and review of the literature. *Clin Nephrol* 2001; **56**: 247–50.<br>- Pereda PiA, Marcos GR, Plaza MV, Gonzales JC, Garcia EG. Complete remission of loiasis-associated nephrotic syndrome with collapsing glomerulopathy after diethilcarbamazine treatment. *Enf Emerg* 2010; **12**: 155–8.<br>- Pérez Pérez AJ, Sobrado J, Cigarrán S, Valdés R, González L, Courel M. [Filariasis *loa-loa* como causa de uremia terminal]. *Nefrologia* 1987; **7**: 183–5.<br>- Malik STA, McHugh M, Morley AR, Ngu J, Qureshi M, Wilkinson R. Filariasis (*Loa loa*) associated with membranous glomerulonephritis: demonstration of filarial antigen. *Kidney interactions* 1981; **20**: 157–157.<br>- Katner H, Eugene Beyt B, Krotoski WA. Loiasis and renal failure. *South Med J* 1984; **77**: 907–8.<br>- Fritel D, Bariety J, Gentilini M. [Loase et néphropathie. Etude anatomoclinique de trois cas]. *Med Afr Noire* 1970; **1**: 13–5.<br>- Cruel T, Arborio M, Schill H, *et al.* [Néphropathie et filariose à *Loa loa*. A propos d’un cas de réaction adverse à la prise d’ivermectine]. *Bulletin de la Société de pathologie exotique* 1997; **90**: 179–81.<br>- Arborio M, Schill H, Neveux Y, *et al.* [Néphropathie et filariose à *Loa loa* traitée par ivermectine]. Thérapeutiques antiparasitaires : communications. 1995; **55**.<br>- Abel L, Joly V, Yeni P, Carbon C. Apheresis in the management of loiasis with high microfilariaemia and renal disease. *Br Med J* 1986; **292**: 24–5.
Loa loa microfilariae in urine	- Barierty J, Barbier M, Laigre M-C, *et al.* [Protéinurie et loase. Etude histologique, optique et électronique d’un cas]. *Société médicale des hôpitaux de Paris* 1967; **118**: 1015–25.<br>- Fritel D, Bariety J, Gentilini M. [Loase et néphropathie. Etude anatomoclinique de trois cas]. *Med Afr Noire* 1970; **1**: 13–5.<br>- Katner H, Eugene Beyt B, Krotoski WA. Loiasis and renal failure. *South Med J* 1984; **77**: 907–8.

Table 2. Prevalence of other comorbidities in the population study

	Total	Level of proteinuria				p-value*
		Negative	Traces	Lightly positive (+)	Highly positive (++ or +++)	
**N. subjects** (n, %)	990	555 (56·1%)	325 (32·8%)	89 (9·0%)	21 (2·1%)	
**Haematuria (n, %)**	990					< 0·0001
Negative or Traces	907 (91·6%)	529 (95·3%)	299 (92·0%)	68 (76·4%)	11 (52·4%)	
Positive	83 (8·4%)	26 (4·7%)	26 (8·0%)	21 (23·6%)	10 (47·6%)	
**Schistosomiasis** (n, %)	98	31	32	23	12	
Presence of eggs (urines)	0 (0·0%)	0 (0·0%)	0 (0·0%)	0 (0·0%)	0 (0·0%)	N/A
Positive serology (plasma)	6 (6·1%)	4 (12·9%)	0 (0·0%)	1 (4·3%)	1 (8·3%)	N/A
**Stools examination** (n, %)	58	24	22	10	2	
Hookworm presence	0 (0·0%)	0 (0·0%)	0 (0·0%)	0 (0·0%)	0 (0·0%)	N/A
Ascaris presence	36 (62·1%)	16 (66·7%)	14 (63·6%)	5 (50·0%)	1 (50·0%)	0·81
Trichuris presence	39 (67·2%)	16 (66·7%)	13 (59·1%)	8 (80·0%)	2 (100·0%)	0·61

* Chi-2 for categorical variable (with Yates correction if 1 $n_{ij} < 5$)
N/A: not applicable

Table 3. Distribution of the main variables of interest according to haematuria status

	Total	Level of haematuria				p-value*
		Negative	Traces	Lightly positive (+)	Highly positive (++ or +++)	
**N. subjects** (n, %)	990	895 (90·4%)	12 (1·2%)	24 (2·4%)	59 (6·0%)	
**Sex-ratio** (M/F)	1·67	1·8	1·4	1·0	0·8	0·010
**Age** in years (mean ± SD)	50·8 ± 14·8	50·5 ± 14·7	61·5 ± 9·8	54·9 ± 16·5	51·6 ± 15·9	0·023
***Loa* MFD status**						0·25
Positive (n, %)	342 (34·5%)	301 (33·8%)6	5 (41·7%)	12 (50·0%)	24 (41·7%)	
***Loa* MFD** (mf/mL)						0·25
Arith. Mean ± SD	2409·4 ± 7398·5	2259·0 ± 7259·6	5036·7 ± 10 118·1	4944·6 ± 10 852·9	3124·7 ± 7069·9	
Median [ITQ]	0 [0-540]	0 [0-440]	0 [0-4615]	120 [0-3735]	0 [0-1160]	
Geom. Mean	1647	1707	1806	3981	2153	
***Loa* MFD categories** (n, %)						0·29
0 mf/mL	648 (65·5%)	594 (66·4%)	7 (58·3%)	12 (50·0%)	35 (59·3%)	
1-4999 mf/mL	217 (21·9%)	194 (21·7%)	2 (16·7%)	7 (29·2%)	14 (23·7%)	
5000-14 999 mf/mL	82 (8·3%)	73 (8·2%)	1 (8·3%)	3 (12·5%)	5 (8·5%)	
≥15 000 mf/mL	43 (4·3%)	34 (3·8%)	2 (16·7%)	2 (8·3%)	5 (8·5%)	

Abbreviations: SD, Standard deviation; MFD, microfilarial density; ITQ, interquartile range; NC, not calculable; Arith, arithmetric; Geom., Geometric.
* Chi-2 for categorical variable (with Yates correction if 1 $n_{ij} < 5$) and Kruskal-Wallis rank test for continuous variables.
